# Revascularisation patterns and characteristics after erythropoietin pretreatment and multiple burr holes in patients who had acute stroke with perfusion impairment

**DOI:** 10.1136/svn-2023-002831

**Published:** 2024-05-30

**Authors:** Seong-Joon Lee, So Young Park, Geun Hwa Park, Jin Soo Lee, Yong Cheol Lim, Ji Man Hong

**Affiliations:** 1 Department of Neurology, Ajou University School of Medicine, Suwon, Korea (the Republic of); 2 Department of Biomedical Sciences, Ajou University Graduate School of Medicine, Suwon, Korea (the Republic of); 3 Department of Neurosurgery, Ajou University School of Medicine, Suwon, Gyeonggi-do, Korea (the Republic of)

**Keywords:** Stroke, Perfusion, Cerebrovascular Disorders, Meninges, Cerebrovascular Circulation

## Abstract

**Background:**

Transdural collaterals, originating mainly from the extracalvarial superficial temporal artery and intracalvarial middle meningeal artery via the external carotid artery (ECA), have been observed after revascularisation surgery. However, the origin of these collaterals in patients with stroke with perfusion insufficiency is not yet known. Therefore, we studied the revascularisation patterns and characteristics based on the origin of these collaterals.

**Methods:**

We employed erythropoietin pretreatment and performed multiple burr holes under local anaesthesia to achieve transdural revascularisation in patients with acute stroke with perfusion insufficiency. After 6 months, we reassessed the transfemoral cerebral angiography to evaluate the revascularisation patterns. The collaterals were categorised into intracalvarial ECA-dominant (originating from the middle meningeal artery), extracalvarial ECA-dominant (originating from the superficial temporal or occipital artery) and balanced groups. We compared various imaging parameters among these groups.

**Results:**

Overall, 87 patients with 103 treated hemispheres were involved. Among them, 57.3% were classified as intracalvarial ECA-dominant, 20.4% as extracalvarial ECA-dominant and 22.3% as balanced. Most of the hemispheres with intracalvarial or extracalvarial collaterals (vs balanced collaterals) showed successful revascularisation (78/80 (97.5%) vs 12/23 (52.1%)), p<0.001). In ultrasonographic haemodynamic changes according to revascularisation pattern, only the intracalvarial ECA-dominant revascularisation was significantly associated with specific changes in ECA blood flow, leading to the conversion to a low-resistance ECA Doppler sonography waveform.

**Conclusions:**

Our findings suggest that intracalvarial ECA-dominant revascularisation plays a crucial role in the formation of transdural collaterals following combined therapy. These distinct changes in ECA haemodynamics can be non-invasively identified through bedside ultrasound studies.

**WHAT IS ALREADY KNOWN ON THIS TOPIC**

- Transdural collaterals originating from the external carotid artery (ECA) have played a significant role in achieving successful revascularisation in patients with stroke with perfusion insufficiency after undergoing revascularisation surgery.

**WHAT THIS STUDY ADDS**

- This research highlights the crucial significance of intracalvarial ECA-dominant revascularisation in the formation of transdural collaterals after erythropoietin and multiple burr hole combined therapy. The study emphasises the angiographic confirmation of this role and showcases the potential to identify related ECA haemodynamic alterations through non-invasive bedside ultrasound studies.

**HOW THIS STUDY MIGHT AFFECT RESEARCH, PRACTICE OR POLICY**

- These findings emphasise the significance of intracalvarial ECA-dominant revascularisation and predict successful revascularisation through non-invasive ultrasound assessment of ECA haemodynamics in patient care, enhancing treatment strategies.

## Introduction

 Transdural collaterals are blood vessels that help protect the brain from insufficient blood flow from intracranial ischaemia, especially in Moyamoya disease.^1^ In previous revascularisation surgeries,^2 3^ these collaterals were believed to mainly come from a branch of the external carotid artery (ECA) called the superficial temporal artery (STA),^4^ which is located outside the skull. Another branch called the middle meningeal artery (MMA), which is inside the skull, was considered less important for these collaterals.

The multiple burr hole (MBH) procedure is a type of surgery that indirectly improves blood flow to the brain.^5^ Initially used to support other surgical techniques, it is now commonly used on its own.^6 7^ This procedure allows the operator to target multiple areas of the brain and has a low risk of perioperative complications. It can be performed using local anaesthesia and does not expose the patient to the risk of hyperperfusion syndrome.^8^ Although there were concerns about the effectiveness of this procedure in promoting stable blood vessel growth through transdural routes, recent studies have shown positive results.^9^,^11^ Some studies have combined the multiple burr hole procedure with an angiogenic substance called erythropoietin (EPO), which helps enhance blood vessel growth from the healthy extracranial milieu, even in patients with acute stroke with cerebral hypoperfusion.^12^

In terms of anatomy, successful revascularisation from the external carotid circulation can occur through branches inside the skull (intracalvarial MMA) or outside the skull (extracalvarial STA or occipital artery (OCA)) from the ECA. However, the specific revascularisation patterns following MBH procedures have not been fully clarified. Therefore, our study aims to evaluate these patterns after MBHs combined with intravenous EPO administration, focusing on the dominance of either intracalvarial ECA or extracalvarial ECA and determining how it affects the extent of revascularisation.

## Materials and methods

### Study population and management

Between 2010 and 2021, we analysed retrospective data from patients with ischaemic stroke who had reduced blood flow to the brain and had recently experienced neurological deficits within 2 weeks. Patients were enrolled based on the inclusion and exclusion criteria in the NIMBUS (NCT02603406, Neovascularization Induced by Mechanical Barrier Disruption and Systemic Erythropoietin in Patients With Cerebral Perfusion Deficits) trial. These patients were part of our earlier trials^13^ that tested a combined treatment involving the use of MBHs and receiving a substance including intravenous EPO (Epokine/CJ HealthCare or Eporon/DONG-A Pharmaceutics, South Korea) before the procedure. The functional and revascularisation outcomes have been previously reported.^9^

**Figure 1:**
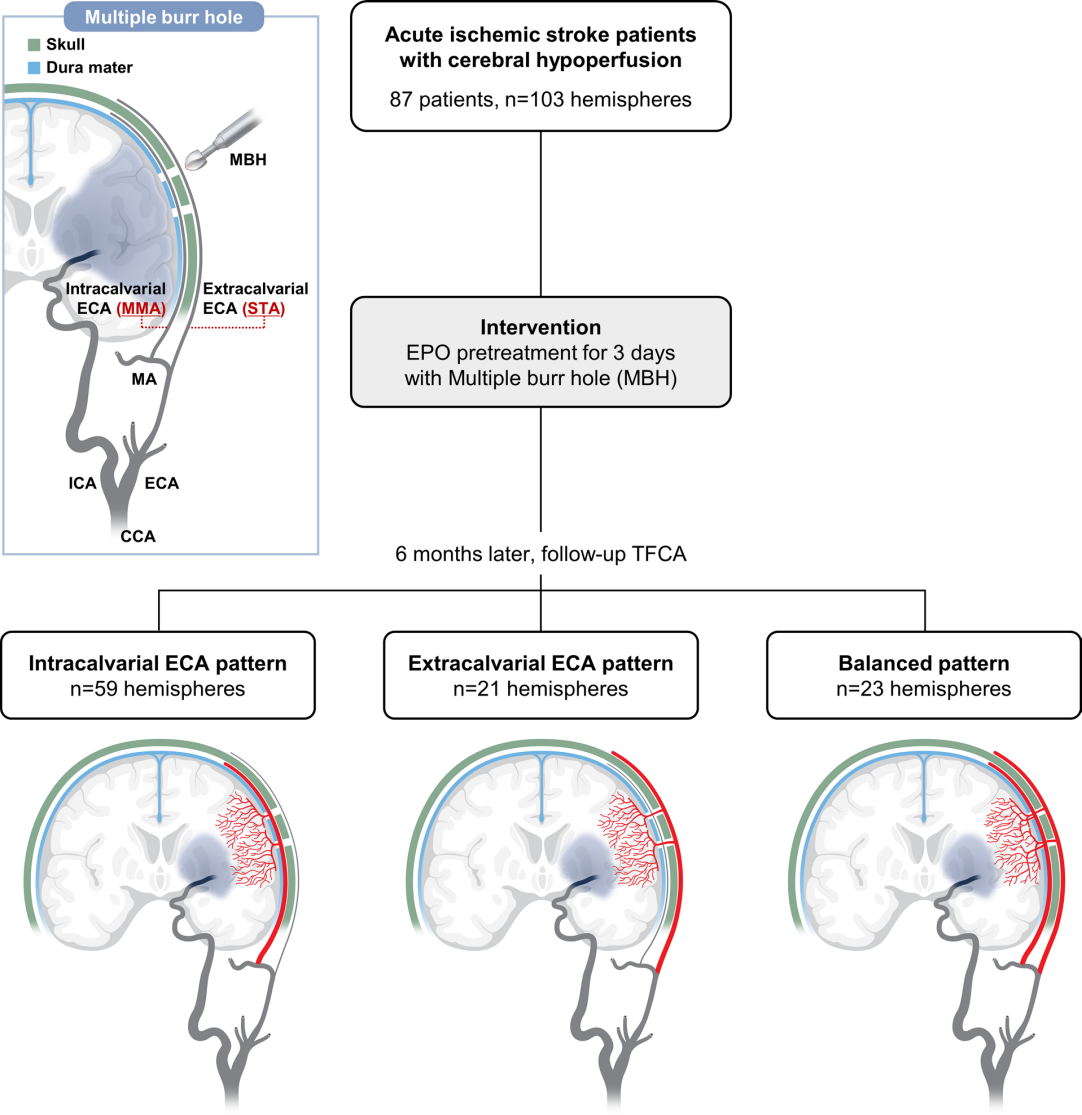
Flowchart of the procedure and subgrouping. CCA, common carotid artery; ECA, external carotid artery; EPO, erythropoietin; ICA, internal carotid artery; MBH, multiple burr hole; MMA, middle meningeal artery; STA, superficial temporal artery; TFCA, transfemoral cerebral angiography.

A total of 87 patients (103 hemispheres) were included in the analysis. The procedure followed the protocol used in our earlier trials (see figure 1). EPO was administered 3 days preoperatively. Local anaesthesia was applied through a lidocaine injection at specified incision points. A scalp incision of 3–4 cm was made for each burr hole, and the burr holes, measuring 2–3 cm each, were created using the Midas Rex high-speed drill. Any bleeding from diploic veins in the bone was addressed using bone wax. A cruciate incision in the dura at each burr hole facilitated the disruption of the mechanical barrier to promote collateral formation. Closure of the scalp incisions was subsequently performed using 2–0 Vicryl sutures and staples. Burr holes were made in areas where blood flow to the brain was not sufficient, and this was done with the patient under local anaesthesia. Based on our initial experience with how much disruption was needed to improve blood flow (see online supplemental figure 1), we opened the protective layer called the dura mater through each burr hole.

After 6 months, we evaluated the revascularisation using transfemoral cerebral angiography (TFCA), CT perfusion images and carotid duplex sonography studies.

We followed the ethical standards outlined in the 1964 Declaration of Helsinki and its subsequent revisions. We obtained informed consent from all the individuals who participated in the study. To access data that supports this study’s findings, you can request it from the corresponding author.

### Patient analysis

We collected clinical information about the study participants from their electronic medical records. We observed the symptoms and signs they represented within 2 weeks and categorised them into different groups, including hemiparesis, dysarthria, cortical signs, visual symptoms, ataxia, dizziness and other manifestations. We also assessed the time between their hospitalisation for the procedure and the 6-month follow-up, and the presence or absence of confirmed lesions on diffusion-weighted MRI scans. To assess the patient’s functional outcome, we used the modified Rankin Scale (mRS) both before and after the procedure to measure their level of disability or independence.

### Hemispheric analysis

The preoperative perfusion status was scored by modifying the method presented in previous studies, incorporating TFCA and MRI results and then dichotomised into good and bad groups.^14^ At the 6-month mark, we analysed the revascularisation patterns in each hemisphere using TFCA images. The patterns were categorised into three types: (1) intracalvarial ECA-dominant (mainly involving the MMA), (2) extracalvarial ECA-dominant (mainly involving the STA or OCA) or balanced (no significant growth or non-specific blush). We determined the dominance based on several factors, including the visualisation of new blood vessel growth through the skull, the enlargement of the arterial diameter in the MMA or STA compared with the initial angiography, or the arrival speed of collaterals to the anastomosis point during the angiographic procedure. In addition to analysing the origin of revascularisation, we also assessed other characteristics of the hemisphere using CT angiography with perfusion parameters. We compared changes in cerebral blood flow (CBF), cerebral blood volume (CBV), time-to-peak (TTP) and mean transit time (MTT) between the baseline and the 6-month follow-up. The extent of revascularisation was determined by analysing lateral projections^15^ of the TFCA images, and it was graded as excellent (≥66% of the affected area revascularised), good (≥33% revascularised), fair (<33% revascularised) or poor (no revascularisation observed).^16^ Successful revascularisation was also defined as a case with excellent, good and fair grades in the ipsilateral hemisphere, except for the absence of revascularisation (none).^13^

### Ultrasonographic haemodynamic analysis

We used a duplex ultrasound with a 12-MHz, linear-array transducer (LOGIQ S6; GE) to evaluate the haemodynamics of blood vessels. This evaluation was done both before the procedure and at the 6-month follow-up. During the ultrasound, we examined various vessels, including the internal carotid artery (ICA) and ECA. The ICA was measured downstream from the bulb, where laminar flow is expected. The ECA flow was measured before the point where it branches off into the superior thyroid artery. The pulse repetition frequency was set to 5 Hz. From these measurements, we obtained information such as the peak systolic flow velocity (PSV), the end-diastolic flow velocity (EDV) and the time-averaged peak mean flow velocity (MFV) of the vessels. We also calculated the pulsatility index (PI), which is derived from the formula (PSV−EDV)/MFV and the resistance index (RI), calculated as (PSV−EDV)/PSV. However, we could not visualise the laminar flow of the ICA, or the patient’s condition was unstable; we did not perform the duplex ultrasound assessment.

### Statistical analysis

Continuous variables are presented as mean±SD, and categorical variables are presented as absolute and relative frequencies. We compared the clinical characteristics, hemispheric profiles, degree of revascularisation and changes in perfusion and duplex parameters based on the different revascularisation patterns. We compared the categorical variables by a χ^2^ or Fisher exact test. According to the nature of the numerical variables, they were compared using the analysis of variance. To compare the changes in perfusion parameters between the initial and the 6-month follow-up period, we conducted a paired t-test across the different revascularisation groups. We used the IBM SPSS Statistics V.22 software (Chicago, Illinois, USA) to conduct the statistical analysis. All p values were two-tailed, and variables were considered significant at p<0.05

## Results

### General characteristics and clinical presentation

Table 1 shows the general demographics, presenting the manifestations and clinical courses. Out of the 87 patients, the majority (54.0%) presented with hemiparesis on admission. The second most common presentation was cortical symptoms, which included issues like aphasia and extinction. Out of all the patients, two (2.3%) had a stroke and four (4.6%) experienced a transient ischaemic attack during hospitalisation. These patients underwent a median follow-up period of 5.2 years, resulting in a median mRS of 1 at 6 months. Among the 87 cases, three (3.4%) were confirmed through MRI. Additionally, deterioration on CT perfusion was observed in 3 out of 71 cases (4.2%), but this deterioration was temporary and subsequently restored.

**Table 1:** Clinical characteristics of the population in this study

	Study population(n=87)
Age	51.8±14.9
Sex, male	40 (46.0%)
HTN	50 (57.5%)
DM	23 (26.4%)
Smoking	21 (24.1%)
Family history	12 (13.8%)
Presenting symptoms	
Hemiparesis	47 (54.0%)
Dysarthria	3 (3.5%)
Cortical symptoms	23 (26.4%)
Visual symptoms	2 (2.3%)
Ataxia	1 (1.2%)
Dizziness	1 (1.2%)
Others	10 (11.5%)
Clinical course during hospitalisation	
Stroke	2 (2.3%)
TIA	4 (4.6%)
Admission NIHSS	2 (0, 5)
Admission mRS	1 (0, 4.0)
Follow-up after discharge	
Total follow-up period (year, IQR)	5.2 (3.1–9.2)
At 6 month mRS (IQR)	1 (0–2)
Recurrence of stroke	3/87 (3.4%)
Deterioration on CTP	3/71 (4.2%)

CTP, CT perfusionDM, diabetes mellitus; HTN, hypertension; mRS, modified Rankin scale; NIHSS, National Institute of Health Stroke Scale; TIA, transient ischaemic attack

### Hemispheric analysis

**Figure 2:**
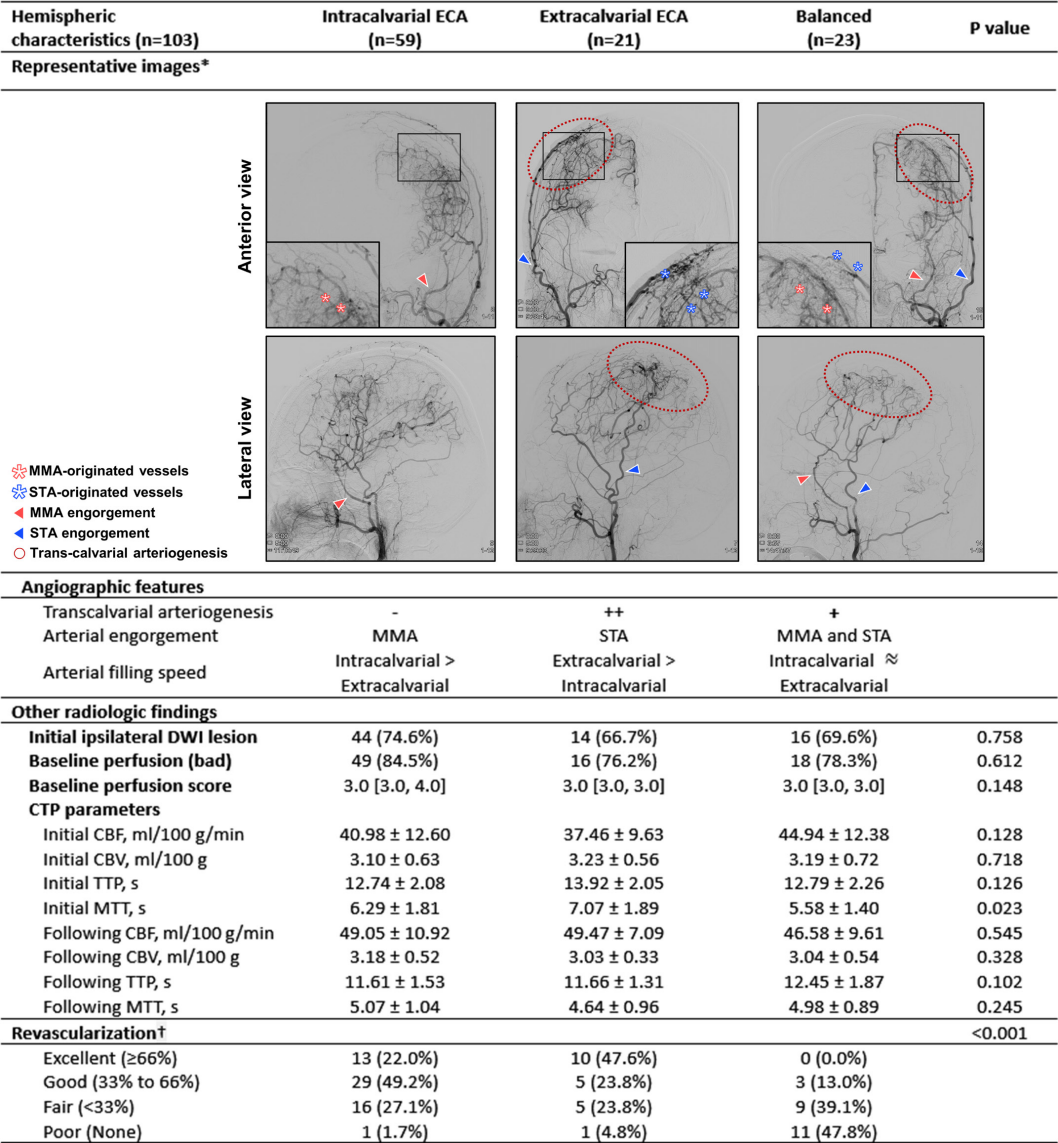
Comparison of the revascularisation patterns of the intracalvarial external carotid artery dominancy and extracalvarial external carotid artery dominancy. *As compared with the extracalvarial ECA revascularisation pattern, there is no transcalvarial arteriogenesis in the intracalvarial ECA revascularisation pattern. †Revascularisation confirmed by transfemoral cerebral angiography after 6 months from multiburr hole operation. CBF, cerebral blood flow; CBV, cerebral blood volume; CTP, CT perfusion; DWI, diffusion-weighted image; ECA, external carotid artery; mRS, modified Rankin Scale; MTT, mean transit time; TTP time-to-peak.

Among the 103 hemispheres, 59 (57.3%) were categorised as intracalvarial ECA-dominant, 21 (20.4%) as extracalvarial ECA-dominant and 23 (22.3%) as balanced. No statistically significant differences were found between the groups in CBF (40.98±12.60 vs 37.46±9.63 vs 44.94±12.38 mL/100 g/min; p=0.128), CBV (3.10±0.63 vs 3.23±0.56 vs 3.19±0.72 mL/100 g; p=0.718) and TTP (12.74±2.08 vs 13.92±2.05 vs 12.79±2.26 s(s); p=0.126), and the preoperative perfusion status score, except for MTT (6.29±1.81 vs 7.07±1.89 vs 5.58±1.40 s(s); p=0.023). However, only the intracalvarial and extracalvarial groups, excluding the balanced group, showed successful revascularisation (58/59 (98.3%) vs 20/21 (95.2%) vs 3/23 (13.0%), p<0.001). There was no statistically significant difference in revascularisation between the intracalvarial and extracalvarial groups (p=0.063) (figure 2).

**Figure 3:**
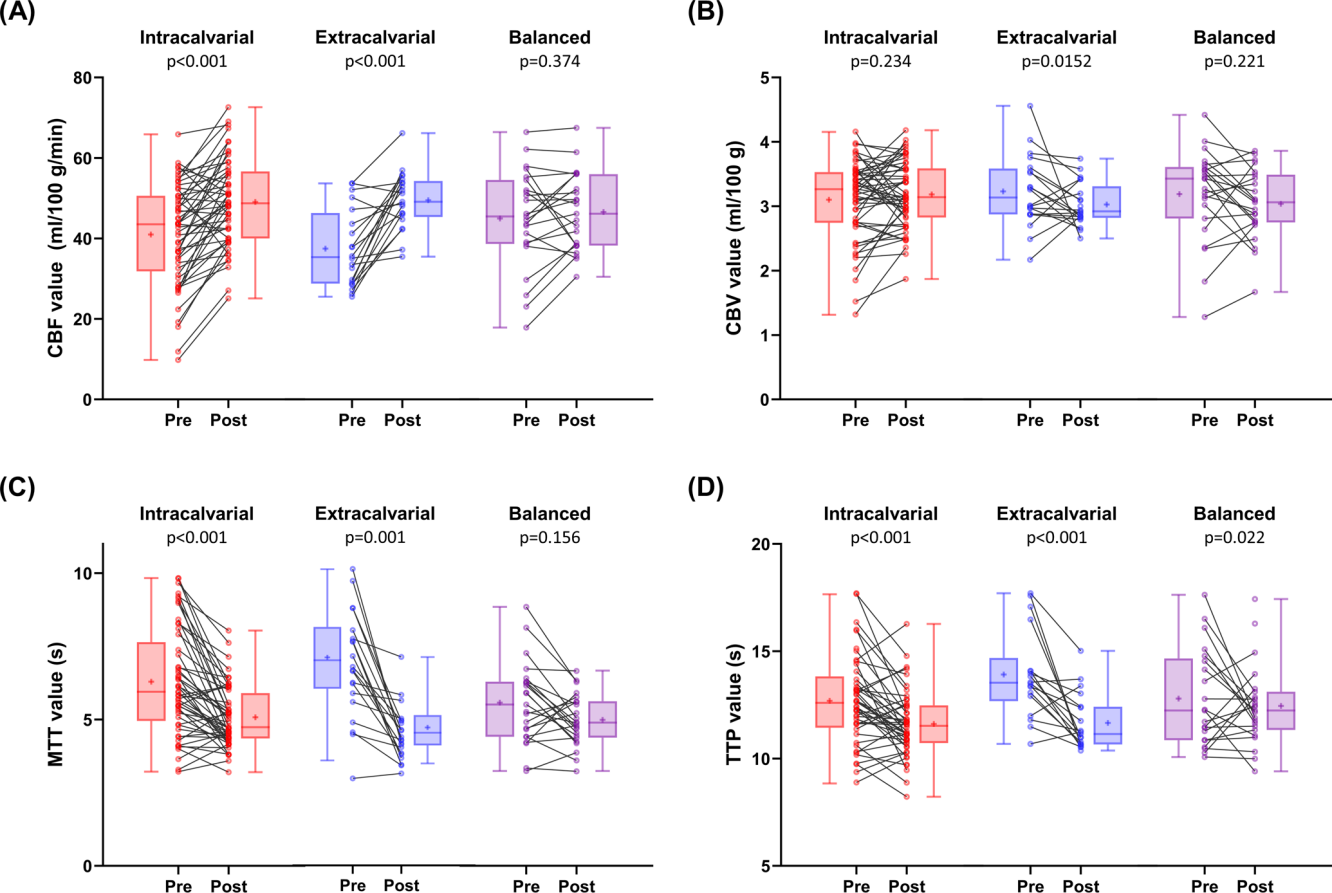
Perfusion changes on the treated hemisphere before and after combined therapy. In comparison to the balanced revascularisation group, both the intracalvarial and extracalvarial revascularisation groups showed significant perfusion changes before and after the combined treatment. CBF, cerebral blood flow; CBV, cerebral blood volume; MTT, mean transit time; TTP, time-to-peak.

Figure 3 showed significant changes in the hemisphere perfusion before and after the combined treatment. The CBF values significantly increased in the intracalvarial (from 40.98±12.60 to 49.05±10.92, p<0.001) and extracalvarial (from 37.46±9.63 to 49.47±7.09, p<0.001) groups. MTT values significantly decreased in the intracalvarial (from 6.29±1.81 to 5.07±1.04; p<0.001) and extracalvarial (from 7.07±1.89 to 4.64±0.96; p<0.001) groups. TTP values significantly decreased in all groups: intracalvarial (from 12.74±2.08 to 11.61±1.53; p<0.001), extracalvarial (from 13.92±2.05 to 11.66±1.31; p<0.001) and balanced (from 12.79±2.26 to 12.45±1.87; p=0.022) groups. When compared with the balanced revascularisation group, both the intracalvarial and extracalvarial revascularisation groups exhibited noteworthy perfusion changes following the combined treatment.

### Ultrasonographic haemodynamic analysis

**Figure 4:**
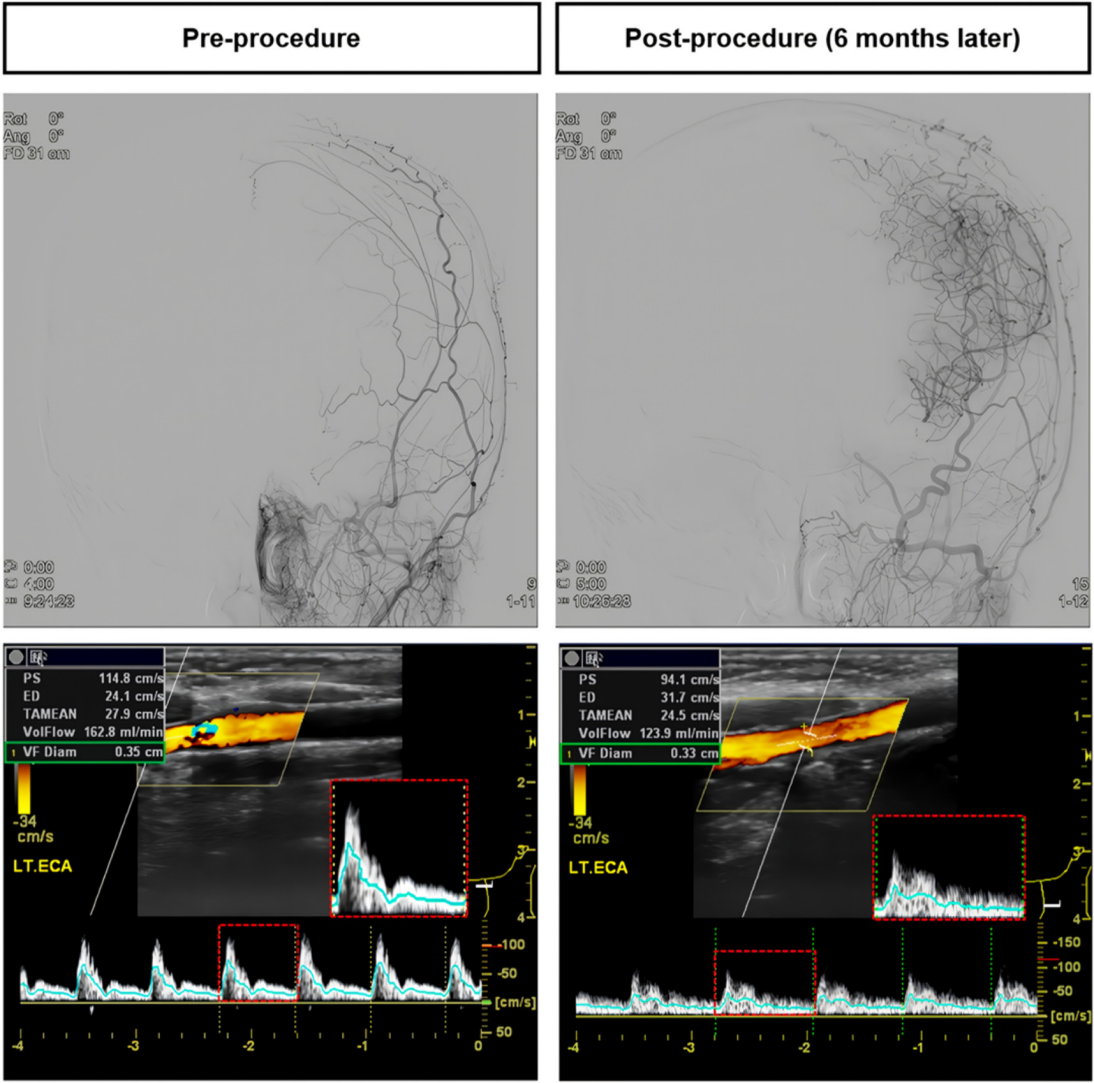
An illustrative case that highlights the haemodynamic changes in the external carotid artery (ECA) during the follow-up period. In this case, the ECA exhibited a transformation to a low-resistance ECA Doppler sonography waveform (indicated by the red box), which is referred to as ‘internalization of the ECA’. This change was observed in conjunction with the intracalvarial ECA revascularisation pattern in the 6-month transfemoral cerebral angiography follow-up. The figure illustrates how the ECA’s blood flow dynamics underwent a significant alteration as a result of the intracalvarial ECA revascularisation process.

When comparing the haemodynamic changes of the ICA and ECA using duplex sonography, a decrease in the PI (from 1.90±0.40 to 1.67±0.33, p=0.001) and RI (from 0.83±0.08 to 0.78±0.08, p<0.0001) was observed in the intracalvarial ECA-dominant hemispheres. However, such changes were not significant in the extracalvarial ECA-dominant and balanced hemispheres (table 2). An example of a representative case from the intracalvarial ECA collateral group is shown in figure 4.

**Table 2:** Ultrasonographic haemodynamic changes according to revascularisation patterns: before and after treatment

Revascularisation patterns(n=70/103 hemispheres)	Haemodynamics of ICA		P	Haemodynamics of ECA		P value
	Pre-procedure	Post-procedure		Pre-procedure	Post-procedure	
Intracalvarial ECA (n=39)						
MAV (cm/s)	26.1±20.1	28.5±17.9	0.230	46.2±14.2	51.5±12.1	0.057
Flow volume (mL/min)	112.4±88.7	110.5±95.1	0.877	163.9±80.0	212.9±88.3	0.005
Pulsatility index	1.35±0.79	1.44±0.82	0.533	1.90±0.40	1.67±0.33	0.001
Resistance index	0.66±0.18	0.69±0.19	0.511	0.83±0.08	0.78±0.08	<0.001
Extracalvarial ECA (n=15)						
MAV (cm/s)	37.9±18.3	33.5±18.5	0.395	51.8±19.4	49.3±15.3	0.584
Flow volume (mL/min)	210.8±164.2	196.3±208.4	0.677	230.6±117.8	181.7±55.4	0.155
Pulsatility index	1.07±0.63	1.29±0.59	0.078	1.72±0.46	1.63±0.45	0.413
Resistance index	0.59±0.16	0.67±0.15	0.043	0.79±0.09	0.77±0.10	0.285
Balanced (n=16)						
MAV (cm/s)	29.1±16.0	29.6±14.3	0.8941	38.0±11.3	40.3±10.2	0.559
Flow volume (mL/min)	134.7±96.4	208.0±238.4	0.199	179.6±77.6	190.3±95.5	0.603
Pulsatility index	1.10±0.25	1.28±0.58	0.305	2.06±053	1.92±0.51	0.300
Resistance index	0.63±0.09	0.66±0.14	0.365	0.86±0.09	0.83±0.09	0.302

ECA, external carotid artery; ICA, internal carotid artery; MAV, mean arterial velocity

## Discussion

The study results indicate that the revascularisation patterns in patients with acute Moyamoya who received multiple burr holes and intravenous EPO can be classified as either intracalvarial or extracalvarial, and the extent of revascularisation is not influenced by whether the primary source of transdural collaterals is located inside or outside the skull. Furthermore, the results of the follow-up ultrasonography revealed a notable reduction in pulsatility within the ECA when the transdural collaterals were formed inside the skull (known as intracalvarial ECA collaterals).

Our study shows that using our combined treatment can improve the development of new blood vessels and successfully restore blood flow through either the intracalvarial or extracalvarial collateral pathway. These findings are consistent with a recent study that also used MBH procedures combined with dural inversion and periosteal synangiosis.^11^ The study found that neovascularisation primarily occurs through the STA and MMA, with less contribution from other arteries such as the posterior auricular artery, OCA and deep temporal artery.

Additionally, our study suggests that the MMA may play a more significant role in achieving long-term revascularisation compared with the STA, which provides more immediate revascularisation through direct bypass surgery.^17 18^ In the future, minimally-invasive techniques may focus on gently disrupting the meningeal layers and improving contact between the brain and the outer periosteal dura layer to promote the formation of new blood vessels.^19^ The critical barrier in the MBH procedure might not be the skull, but rather the dura mater (online supplemental figure 1). After breaking this barrier, the administration of EPO can effectively reduce inflammation and facilitate the maturation of blood vessels.^12^ Promoting the repair of the meningeal vasculature is also crucial, and one potential approach is to recruit wound-healing macrophages from the peripheral blood. This strategy has shown promise in models of mild traumatic brain injury, and it may contribute to effective vascular repair in the meninges.^20^

In the current study, the reductions of the PI and RI in the ECA on duplex sonography were associated with intracalvarial-originated ECA revascularisation via the middle MMA. This resulted in a low-resistance Doppler waveform in the ECA, similar to what is seen in a normal ICA.^21^ Such conversion to a low-resistance Doppler sonography waveform in the ECA has been termed ‘internalization’, as it mimics the spectral tracings in a normal ICA. However, such specific haemodynamic alterations were not related to extracalvarial-originated ECA revascularisation. These findings are consistent with previous studies that have observed decreased RI in intracalvarial dural arteriovenous fistulas,^22^ where the main arterial supply comes from meningeal vessels. However, we did not find these specific haemodynamic changes in cases where the ECA revascularisation originated from outside the skull. Nonetheless, duplex sonography performed after the procedure can help identify the haemodynamic origin of ECA collaterals in a non-invasive manner.

Our interpretations can be limited by the fact that only visual grading of arteriogenesis dominancy was performed. Quantitative methods were not used, and thus, a complete dichotomy of dominancy could not be performed. Nonetheless, only a small number of cases demonstrated a complete absence of the extracalvarial or intracalvarial ECA, and most exhibited revascularisation to some degree from both the extracalvarial and intracalvarial ECA. Despite different revascularisation patterns, there was no difference in the degree of revascularisation; therefore, our results may be justified in this regard.

In conclusion, both the intracalvarial and extracalvarial ECA collaterals can result in successful revascularisation after combined therapy comprising MBH revascularisation with EPO for Moyamoya. ECA PI and RI changes may predict intracalvarial versus extracalvarial ECA-originated revascularisation patterns.

## supplementary material

10.1136/svn-2023-002831 online supplemental file 1

## Data Availability

The datasets in the current study are available from the corresponding author on reasonable request.
