# Supplementary figures and images for: Revascularisation patterns and characteristics after erythropoietin pretreatment and multiple burr holes in patients who had acute stroke with perfusion impairment
Source: Stroke Vasc Neurol. 2024 May 30;10(1):e002831. doi: 10.1136/svn-2023-002831 (PMC11877436; doi:10.1136/svn-2023-002831)

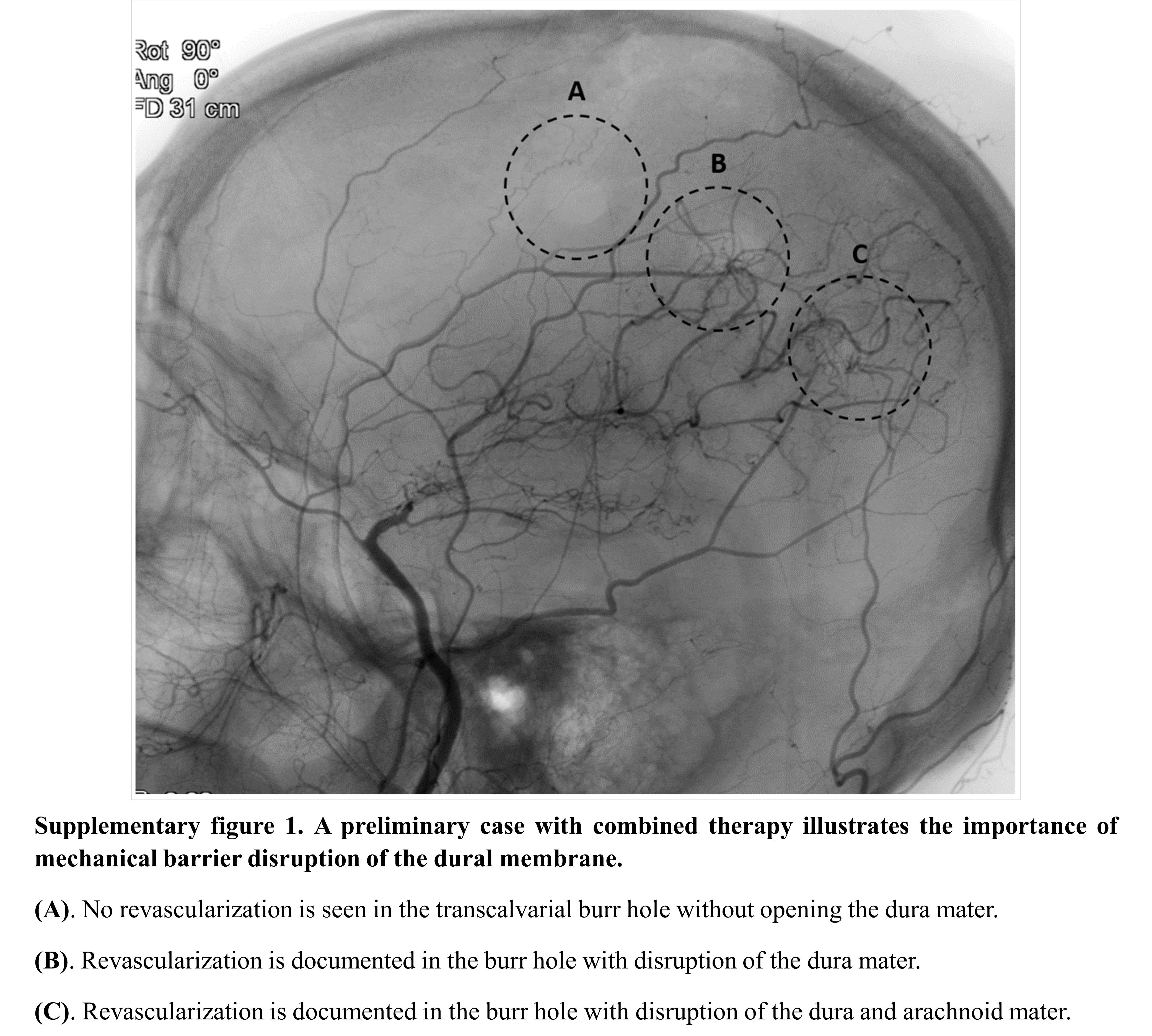

Supplement: online supplemental file 1 [file svn-10-1-s001.jpg]
